# Impact of a training strategy on improving compliance of hand hygiene and gloving during the placement of a short peripheral venous catheter: the multicentre study CleanHand4

**DOI:** 10.1186/s12909-023-04727-x

**Published:** 2023-10-06

**Authors:** Mathilde Farizon, Sandra dos Santos, Lucas Richard, Agnès Petiteau, Anne-Sophie Valentin, Nathalie van der Mee-Marquet, on behalf of the CleanHand4 collaboration group, Kimberley Alexandre, Alexandra Allaire, Karine Amandier, Nathalie Audrain, Amina Azzam, Mehdi Bastard, Mélika Berrahal, Yasmina Berrouane, Marie-Camille Betti, Claire Bianchi, Mathilde Blanié, Laetitia Borrelys, Caroline Boschet, Alexandre Bourdet, Jihane Brisson, Anne Brechat, Dominique Buiguez, Sandra Caffort, Céline Chatelet, Catherine Chatin, Karine Chevalier, Armelle Choquet, Amélie Coire, Karine Courcelle, Nathalie Cremoux, Michèle Dangel, Cécile Debarre, Lydia Decruyenaere, Peggy Delavault, Frédérique Diaw, Marie Dobras, Carole Domrault-Tanguy, Sylvie Drot, Audrey Duchemin, Isabelle Durand-Joly, Claude El Kallas, Christelle Formery, Pierre Fournier, Aline Franck, Blanche Ghalloussi, Nathalie Ghironi, Marie Godet, Anne Goudouneche, Jill Gregoire, Hedia Guermazi, Nadia Idri, Emmanuelle Jacques–Gustave, Sylvie Joron, Laurence Joseph, Anne-Marie Kayoulou-Bour, Maha Keswani, Annick Kmiecik, Marie Lafargue, Magalie Laffon, Elodie Lafond, Bruno Le Falher, Cécile Le Gouil, Sophie Leconte, Florence Malfondet, Sandrine Marty, Nadine Mertel, Virginie Morange, Floriane Morette, Jennifer Mouronval, Sylvie Moutarde, Nadine Negrin, Dominique Ollivier, Anne Perez, Pauline Pommier, Kahina Pouponnot, Isabelle Pouy Berlemont, Samantha Raumel, Patricia Rossi, Noella Roudaut, Bénédicte Roux-Sibillon, Dominique Saez de Ibarra, Muriel Schrevens, Ousseini Sidikou, Bertille Tamburro, Catherine Theaude, Sarah Thevenot, Jessica Turpin, Morgane Valsaque, Séverine Veja, Lucie Wojciechowski, Laure Zangoli

**Affiliations:** Cpias Centre Val de Loire, Hôpital Bretonneau, CHRU, Tours, 37044 France

**Keywords:** Short peripheral venous catheters, Asepsis, Hand hygiene, Gloving, Improvement of practices

## Abstract

**Background:**

Patients who have short peripheral venous catheters (PVC) face an elevated risk of developing bloodstream infections. Preventing catheter-related infections relies on implementing multiple measures, including practicing proper hand hygiene (HH) during catheter placement.

**Methods:**

We conducted a four-part study: (1) an evaluation of HH practices through direct observation of PVC placements, coupled with the study of the microbial flora of the HCWs fingers just before the placement; (2) the development of an educational tool based on the collected observational and microbiological data; (3) the training to the HCWs observed during the first part, using this tool; and (4) the subsequent observation of the trained HCWs to measure the impact of the training on practice improvement.

**Results:**

Compliant HH was observed in 23.5% of the 647 HCWs observed during PVC placement before training. The microbiological study revealed fewer pathogens on the fingertips of the HCWs practicing compliant HH compared other HCWs (2.6 vs 11,7%; *p* = 0.003). The comparison of practices before and after training, assessed among 180 HCWs, showed an increase in the proportion of HCWs performing compliant HH (25.0 vs 63.2%; *p* < 0.001).

**Conclusions:**

Training HCWs using our educational tool, which combines reminders of best practices and risk factors associated with PVC-related infections, engaging HCWs (presentation of practice evaluation), identifying professionals deviating from best practices (simulation videos), and objectively assessing fingertip contamination (microbiological study), significantly improved compliance with HH gestures and glove usage. We encourage infection control teams to utilize this tool to raise awareness among HCWs responsible for PVC placement about the risk of infection associated inadequate hand hygiene.

**Supplementary Information:**

The online version contains supplementary material available at 10.1186/s12909-023-04727-x.

## Introduction

The placement of short-term peripheral intravenous catheters (PVCs) is a common medical procedure, estimated at around 25 million cases in France in 2005 [1]. PVC-related infections can have repercussions on patient care, leading to extended hospital stays, delayed treatments, and higher hospitalization costs [2].

PVC-related infections occur as a result of catheter contamination and the factors favouring these infections are multiple [3, 4]. PVC are primarily contaminated by bacteria from the patient’s skin flora if the antisepsis at the insertion site is suboptimal, or from the HCW’s flora when strict asepsis is not observed during PVC placement.

Preventing PVC-related infections relies on implementing a bundle of measures during PVC placement, including hand hygiene (HH) [2, 5–10]. HCWs should have clean hands from the beginning to the end of catheter placement, which requires two HH practices: the first, before preparing the equipments, aimed at eliminating microorganisms present on the HCW’s hands and collected during care provided to other patients; and the second, just before catheter insertion, aimed at eliminating microorganisms collected during patient setup and preparation of the insertion site.

Numerous studies consistently show that HCWs often do not adequately adhere to HH opportunities and technical compliance, with rates generally below 50% [10, 11]. Low HH rates before aseptic procedures pose patient safety risks. While some studies have reported low moment-specific compliance before clean/aseptic procedures, none have focused on PVC placement [12, 13]. Thus, our primary study goal was to assess HH practices during PVC placement and identify discrepancies between optimal and actual compliance.

Interventions aimed at improving HH in patient care are abundant [14], but data concerning HH during catheter placement are scarce [15–17]. In two studies, the implementation of an educational program emphasizing HH led to reduced rates of CVC-related bloodstream infection [15, 16].

While HH procedures are straightforward, improving their implementation by HCWs is a complex challenge [13]. Numerous strategies have been designed to enhance HH, but their effects are often modest [13]. inadequate HH practices are not primarily due to a lack of knowledge or information [18]. Factors such as high workload and limited access to HH facilities have emerged as major contributors to non-compliance with HH guidelines, while subjective norms and attitudes toward HH have been linked to compliance with HH [10].

Many tools are available for promoting HH [14, 19–21]. Behavioral determinants include knowledge, awareness, action control, behavioural facilitation, social influence, attitude, self-efficacy and intention [22–24]. Finally, intervention strategies to promote HH should be iterative and multimodal, involving at least education, system changes and motivation [10, 11, 22, 25]. One study we found introduced a multimodal intervention specifically designed to enhance HH during PVC placement [17]. This intervention consisted of five elements, including teaching sessions, dummy training, e-learning tool, tablet and poster aids, and direct feedback. Prior to the intervention, the HH rate was 11.6% before patient contact, compared to 57.9% after the intervention (*p* < 0.001), and 0.5% before PVC insertion, compared to 45.5% after intervention (*p* < 0.001). However, this study appeared to require a significant time commitment in the field, considering the current workload burdens in clinical services and the need for repeated interventions.

To facilitate behaviour change, HCWs must recognize the risk and comprehend the mechanisms of microorganism transmission during patient care [13, 14]. In healthcare setting, hands can become colonized by pathogenic species [26–30]. Our study’s second objective was to provide direct microbiological evidence of bacterial contamination on HCWs’ hands immediately before the placement of PVC when HH during this procedure is suboptimal. To achieve this goal, we combined the observation of HCWs’ HH practices with a study of the microbial flora on their fingertips just before PVC placement.

To begin, we developed an educational tool tailored for the context of PVC placement. This tool was intended for use during concise educational sessions lasting less than 30 min. It incorporated data acquired from observations and microbiological analyses, complemented by instructional videos showcasing a range of PVC placement scenarios, encompassing both correct procedures and instances with errors. We conducted observations of HCWs both prior to and following their training with our tool to assess its effectiveness in improving hand hygiene.

## Materials and methods

### Context and study design

Since 2019, the French Ministry of Health has mandated that all hospitals in France require their local infection prevention teams to execute the 2022–2025 National Strategy for Infection Prevention and Antibiotic Resistance. Within this initiative, the reduction of catheter-related infections is a primary focus. The national SPIADI network’s role is to aid local infection prevention teams in implementing catheter-related infection monitoring and prevention within their respective facilities. In this context, the SPIADI team extended invitations to all infection control teams from French hospitals to participate in the study. The study, conducted from January to September 2022, consisted of four phases: (1) an evaluation of HH practices was conducted in each participating center, with direct observation of HCWs placing PVCs and the study of the microbial flora present on the fingers of the observed HCWs; (2) the national-level development of an educational tool was conducted using the data obtained during the first phase, including the results of the HH practices evaluation and microbiological data; (3) the local infection control teams trained the HCWs observed during the first phase using the tool; and (4) a second observation of HH practices was conducted by the local infection control teams. This second evaluation aimed to measure the impact of the training on the improvement of HH practices. Comprehensive details regarding the study protocol and participation guidelines can be found in a downloadable technical guide accessible on the SPIADI national network’s website (spiadi.fr).

### Observational study

At each participating center, the infection control team leader was tasked with selecting the ward(s) in which to carry out the study; The recommendation was to choose a specific entity (such as a hospital, department, or unit) and observe graduate or student HCWs placing a PVC in adult patients within that geographical entity. The observations were conducted during the first phase for eight different HCWs. If the number of professionals responsible for PVC insertions within that geographical entity exceeded eight, it was suggested to select the professionals for observation randomly. The observations were conducted during the fourth phase for all the HCWs observed during the first phase and trained during the third phase, with a minimum of 2 months between the second observation and the training. The observations were conducted using a standardized grid examining HH at the beginning of the procedure and immediately prior to PVC insertion (presence/absence of action, type of HH (hand washing or alcohol-based hand rubbing), presence/absence of prerequisites (exposed forearms, short nails, no jewelry) and conformity of the gesture if applicable), and glove use (presence/absence of gloves and time of gloving if applicable), and skin preparation (skin cleansing, antiseptic solution used, spontaneous drying). Supplementary Figure 1 displays the grid. The observation sheets were sent to the national level for analysis. The results were firstly used for the initial evaluation of practices. Secondly, the practices of the HCWs observed before and after training were compared to measure the impact of training on HH compliance. In this study, only the data related to HH and glove use are presented. Comprehensive findings pertaining to the insertion process, including the selection of antiseptics for site preparation, the type of dressings utilized, compliance with antiseptic contact duration, HCW attire, and patient attire, are documented in the 2022 SPIADI national report, which primarily centers on the evaluation of catheter insertion practices performed in 2022. This report is available in French and can be downloaded from the SPIADI network’s website (https://www.spiadi.fr/results?tab=0).

### Microbiological study

During the first phase, the local infection control teams used sterile systems (Amies transport media, Mast, Copan-Brescia, Italy) to swab the fingertips and palm of the hands of the HCWs immediately prior to PVC insertion, following a standardised procedure. The swabs were stored at room temperature and sent to the national laboratory. Upon receipt, they were labeled and paired with observation sheets, and then inoculated onto Trypticase Soja sheep blood agar (bioMérieux, Marcy l’Etoile, France). All visible microbial colonies after a 48-h incubation at 37 °C under aerobic conditions were identified using MALDI-TOF technology (Bruker Daltonics, France). *S. aureus*, *Enterobacteriales* and *Enterococci* were tested for antibiotic susceptibility according to French guidelines [31]. The microbiological data firstly provided an overview of the contamination of the fingers of the HCWs just before PVC placement. Secondly, the microbiological data, along with the data obtained from the observation of practices, were entered into an Excel table to investigate the correlation between the presence of pathogens on the fingers and the level of compliance of HH among the HCWs.

### Design and use of the educational tool

A training tool was developed by the national team, underwent testing by local teams before the final version was delivered. The tool was introduced to the local teams during a web conference, where they had the opportunity to ask any questions. A technical guide accompanied the tool to assist with its usage. The tool is available for download on the national network’s website (https://www.spiadi.fr/tools?tab=1). The local infection control teams then trained the HCWs observed during the first phase of the study using the national tool. For the study, a single session may have been conducted in each hospital, with consideration for the acceptability of the study by the local infection control teams. An evaluation of the acceptability of the tool was conducted using a questionnaire intended for trained HCWs and trainers.

## Results

### Initial evaluation of practices

A total of 91 French hospitals participated in the first phase of the study: six university and regional hospitals, one military hospital, 38 general hospitals, 37 short-stay clinics, three home care centers, two oncology centers, two rehabilitation centers, one long-stay hospital and one haemodialysis center (Supplementary Figure 2). The number of observations varied between 3 and 9 depending on the center (median 8). A total of 647 HCWs were observed, of whom 90% (*n* = 581) were nurses, 6% radiographers (*n* = 40), 2% were doctors (*n* = 10), and 1% were midwives (*n* = 8). For 8 cases, the status was not documented (1%). The HCWs were very diverse and distributed as follows: 62% in short-stay medical units, 25% in surgical departments, 8% in long-stay care units, and 5% in intensive care units. Considering that the insertion of PVCs should meet the same standards regardless of the department, we did not conduct an analysis of observation results based on the HCWs’ origins.

Out of the 647 HCWs observed, 23.5% (*n* = 152) performed the two expected HH gestures in a compliant manner (Table 1). Among the 495 remaining HCWs, 53.1% (*n* = 263) performed a single compliant HH, while 46.9% (*n* = 232) did not comply with either of the 2 HH gestures. HH compliance was higher for the first HH (i.e., the one performed at the beginning of care) than for the second one (i.e., the one performed just before catheter placement) (390 (60.3%) vs 177 (37.3%); *p* < 0.001). For the first opportunity, HH was not performed at all in 10.8% of cases (*n* = 70), and for the second opportunity, this omission was observed in 45.9% of cases (*n* = 297) (*p* < 0.001).

**Table 1:** HH compliance^a^ observed for the 647 HCWs (%)

Observation of HH	No compliant HH				One compliant HH				Two compliant HH	All
1^rst^ HH gesture	ND	ND	NC	NC	ND	NC	C	C	C	390
2^nd^ HH gesture	ND	NC	ND	NC	C	C	ND	NC	C	177
N HCWs	50	7	87	88	13	12	160	78	152 (23,5)	647
	232 (35,9)				263 (40,6)					

ND Not performed; NC Performed but non-compliant HH; C Compliant HH

^a^According to French national guidelines, HH is considered to be performed but not compliant if any of the prerequisites are not met or if the technique is not appropriate. The prerequisites are as follows: Professional attire with exposed forearms, short and clean nails without polish, gel, artificial nails, or resin, no jewelry on hands and wrists (ring, wedding ring, watch, bracelet), and dry and visibly unsoiled hands.The technique should include the recommended steps, and the rubbing should continue until the hands are completely dry (for a duration of 20 to 30 s)

The hands of HCWs must be protected from accidental blood exposure during catheter insertion. To achieve this goal without compromising HH, gloves should be donned after the second HH gesture. The observations revealed that 22.7% of the HCWs (*n* = 147) gloved their hands at the correct time (Table 2). The remaining HCWs placed the PVC without gloves (275; 42.5%) or gloved their hands too early (225; 34.8%).

**Table 2:** Gloving compliance observed for the 647 HCWs (%)

Observation of gloving	No compliant HH (*n* = 232)	One compliant HH (*n* = 263)	Two compliant HH (*n* = 152)	All (*n* = 647)
No	95 (40.9)	100 (38.0)	80 (52.6)	275
Non-compliant	96 (41.4)	129 (49.0)	0	225
Compliant	41 (17.7)	34 (12.9)	72 (47.4)	147

The remaining HCWs (34.8%; *n* = 225) gloved too early (i.e., either before starting to prepare the material (5.7%; *n* = 37) or before carrying out the skin preparation (29.1%; *n* = 188)), and missed the second HH opportunity. Gloving compliance was higher among HCWs who performed two compliant HH compared to those who performed one or none in a compliant manner (72 (47.4%) vs 75 (15.1%); *p* < 0,001).

### Contamination of the fingers of the HCWs just before PVC placement

Each observation of PVC placement led to a microbiological sampling of the HCWs’ fingertips (gloved or not). Out of the 647 swabs analysed, 9.6% (*n* = 62) revealed the presence of at least one pathogen (one pathogen (*n* = 56) or more (*n* = 6)), and 42.0% (*n* = 272) showed a culture of various microorganisms, mainly skin organisms, such as non-*aureus Staphylococci* and *Micrococci*, without any presence of pathogenic bacteria. Among the 334 swabs exhibiting visible microbial growth, 75.0% (*n* = 251) presented at least one bacterium commonly found in the skin flora. The most common species were coagulase-negative *Staphylococci* (*n* = 126), *Micrococcus* (*n* = 63), *Moraxella* (*n* = 55) and *Corynebacterium* (*n* = 18). Additionally, 43.7% (*n* = 146) of the swabs contained at least one environmental microorganism such as *Bacillus* species not belonging to the *cereus* complex (*n* = 76), *Paenibacillus* (*n* = 20), *Brevundimonas* (*n* = 17) and *Brachybacterum* (*n* = 13). Among the 71 identified pathogens, the predominant ones were *Acinetobacter* (*n* = 27; 38.0%), 13 *Bacillus* belonging to the *cereus* complex (18.3%), ten *Enterobacteriales* (14.1%) including one *Enterobacter cloacae,* one *Proteus mirabilis,* one *Serratia proteamaculans,* one *Ewingella americana* and six *Pantoea*, eight *Enterococci* (11.3%) including six *E. faecalis*, two *E. faecium*, eight *S. aureus* (11.3%), two *Stenotrophomonas* (2.8%) and three yeasts (4.2%). Among the *S. aureus* isolates, six had a wild phenotype, one was an erythromycin and fluoroquinolone resistant MSSA, and one was a fluoroquinolone resistant MRSA. The *Enterobacteriales* were susceptible to third generation cephalosporins and carbapenems, and the *Enterococci* were susceptible to vancomycin. The pathogens identified did not vary based on the number of compliant HH performed prior to swabbing.

We investigated the correlation between the presence of pathogens on the fingers and the level of HH compliance among HCWs before PVC placement, and found a lower presence of pathogens on the fingertips of HCWs who performed 2 compliant HH gestures in a compliant manner compared to other HCWs (2.6% vs 11.7%; *p* = 0.003) (Table 3). For the HCWs who performed 2 compliant HH gestures, the presence of pathogens on the fingers was not influenced by the use of gloves (*p* = 0.539). By contrast, for the HCWs who did not perform two compliant HH, we found a higher presence of pathogens on the fingertips of HCWs who did not glove compared to other HCWs (16.9% vs 8.3%; *p* = 0.004).

**Table 3:** Pathogen detection according to HH compliance observed for the 647 HCWs

Observations	No compliant HH (*n* = 232)	One compliant HH (*n* = 263)	Two compliant HH (*n* = 152)	All (*n* = 647)
Swabs with ≥ 1 pathogen	25 (10.8)	33 (12.5)	4 (2.6)	62 (9.6)
Without gloves	14/95 (14.7)	19/100 (19.0)	1/80 (1.2)	34/275 (12.4)
With gloves	11/137 (8.0)	14/163 (8.6)	3/72 (4.2)	28/372 (7.5)

### Training

A training tool was developed at the national level and distributed to local teams. The tool includes a user guide that outlines the topics to be covered during the training session, and a slideshow that presents the following topics in a specific order: (1) Reminder of the importance of PVC-related infection prevention, (2) current guidelines for HH during PVC placement, with a particular focus on the necessity of performing two HH gestures, (3) results of the practice evaluation and microbiological study conducted in Phase 1, (4) correlation between the level of finger contamination among HCWs and HH compliance, (5) three short videos: the first two videos illustrating common inappropriate situations observed (i.e., PVC placement without proper HH, and PVC placement with compliance for the first HH but premature gloving), and the third video showing proper PVC placement with both HH performed, and (6) a debriefing framework designed to ensure that training participants have a thorough understaanding of the mechanisms of finger contamination during CVP placement when HH is not adequate. A total of 48 local teams conducted on-site training sessions using the provided tool for the HCWs observed during the first phase. An evaluation of the tool’s acceptability was conducted using a questionnaire administred to trained HCWs and trainers. The analysis of the questionnaires completed by 48 trainers and 280 trained HCWs at the end of the training, revealed an average training duration of 70 min (15–180), divided into an average training time of 44 min (10–90) and an average discussion time of 26 min (5–90). Trained HCWs expressed a strong interest in the microbiological results (mean score of 8.5/10) and the videos (mean value 7.7/10). They reported a willingness to change their practices (mean 8.2/10) and recommend the training (mean 8.8/10). The duration of the training was considered satisfactory, as was the duration of the discussion with the trainer. For the trainers, the training helped them better understand the challenges faced by HCWs in carrying out the two HH gestures at the right time (mean value 7.1/10). They appreciated the educational tool (8.4/10) and the technical guide (8.6/10) and expressed readiness to use the tool again (mean 8.5/10).

### Impact of the training on the improvement of HH and gloving

**Fig. 1:**
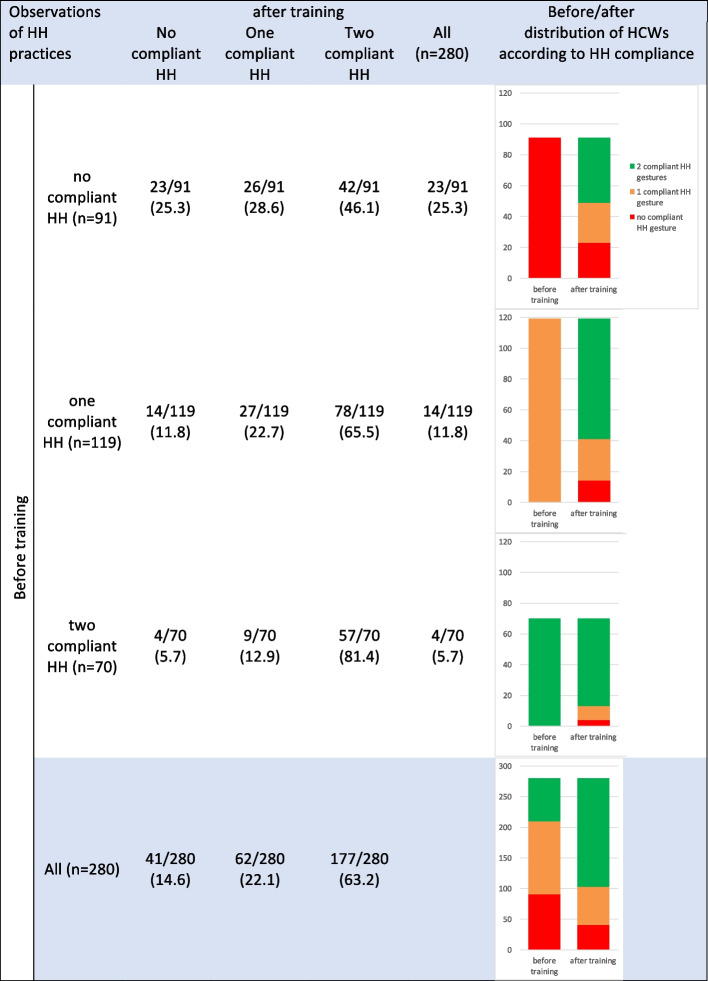
HH compliance of the 280 HCWs before and after training according to initial HH compliance

**Fig. 2:**
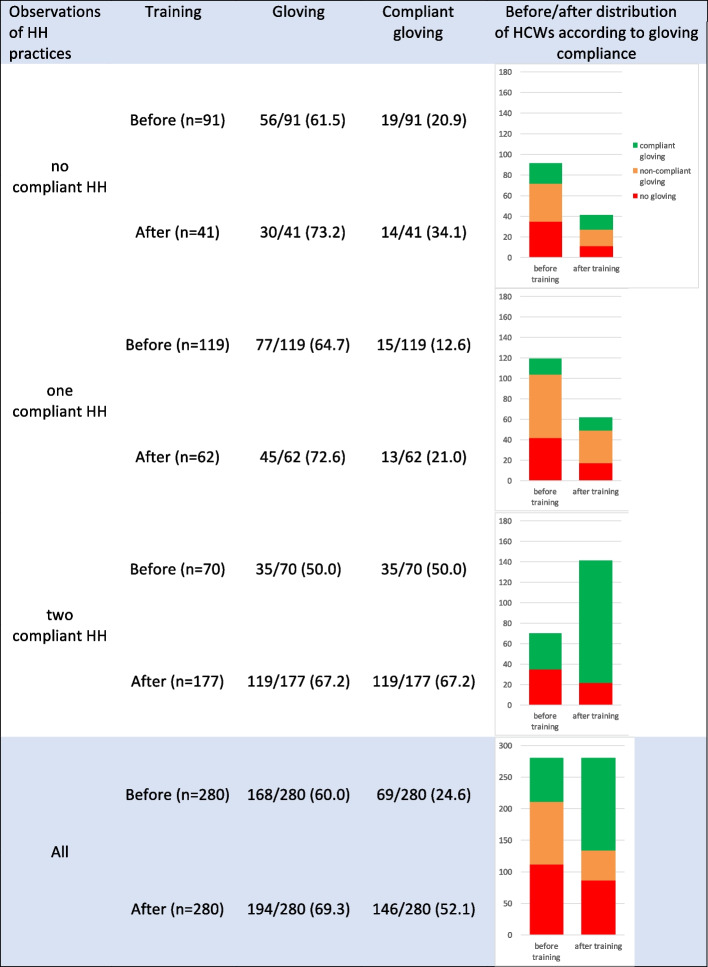
Gloving compliance (%) of the 280 HCWs before and after training, according to initial HH compliance (first phase)

In total, 48 centers participated in both first and fourth phases of the study, allowing for a comparison of the results of practice observations before and after the training for the 280 HCWs in these centers. HH compliance was higher after the training, with 63.2% (*n* = 177) of the 280 HCWs performing the two expected HH gestures, compared to only 25.0% (*n* = 70) of these HCWs before the training (*p* < 0.001) (Fig. 1). The highest progression in HH compliance was observed among HCWs who performed one compliant HH gesture before training, with 65.5% of them performing two HH gestures in a compliant manner after training. Gloving compliance significantly improved after the training, with 52.1% (*n* = 146) of the 280 HCWs donning gloves immediately before PVC placement, compared to 24.6% (*n* = 69) before the training (*p* < 0.001) (Fig. 2). Among HCWs who performed two compliant HH gestures, gloving compliance was even higher, with 67.2% of them correctly donning gloves after the training (*p* < 0,001).

The 280 HCWs followed in Phase 4 and the 367 lost to follow-up after Phase 1 performed the two expected HH gestures in the same proportion (25.0% vs 22.3%; *p* = 0.430). Furthermore, the proportion of HCWs who donned gloves immediately prior to PVC placement was similar in both populations (24.6% vs 21.2%; *p* = 0.308). Therefore, we considered the 280 HCWs observed before and after the training to be representative of the entire population of HCWs observed in Phase 1.

## Discussion

Our study, carried out in 91 diverse French hospitals, brought new data over HH during the placement of a PVC.

Rigourous antisepsis during catheter placement, particularly proper HH, is a well-known factor contributing to the prevention of catheter-related infection [1–7]. By conducting direct observations of a substantial number of PVC placements, we have identified a concerning trend in HH practices. Three out of four HCWs fail to perform the two necessary compliant HH gestures during the procedure. Enhancing HH during PVC placement is a crucial priority in the prevention of PVC-related infections.

While studying HH practices, we observed the use of gloves among HCWs erforming PVC insertions. It was found that one in three HCWs donned gloves prematurely, either at the start of the procedure or before applying the antiseptic. Consequently, in theses cases, HCWs inserted the PVC without conducting the second HH gesture. These data demonstrate how wearing gloves can be a major barrier to implementing HH guidelines, as previously suggested [17, 32]. Considering the significance of this barrier, efforts to improve HH should fully address the issue of glove usage and its interaction with HH practices.

To our knowledge, there is a lack of recent data demonstrating how the hands of HCWs performing PVC insertions become contaminated throughout the procedure when HH practices are suboptimal. We conducted a study examining the microbial flora present on the fingertips of observed HCWs immedately before PVC insertion. The major pathogens associated with catheter-related infections such as *S. aureus*, *Enterobacteriales****,*** and *Enterococci*, were found on the fingertips of one in ten HCWs. Combining the microbiological data with direct observation of HCWs, we found that the presence of pathogens on the fingertips was influenced by the number of compliant HH gestures performed during the procedure, regardless of glove usage. Our findings emphasize the importance of performing two compliant HH gestures, the first before material preparation and the second just before PVC insertion, to ensure clean fingers during catheter insertion. However, zero risk cannot be achieved, as evidenced by the presence of pathogens on the fingers of four HCWs who performed the two compliant HH gestures. These data show that HH is just one of a bundle of preventive measures for catheter-related infections. Several hypotheses can be proposed to explain finger contamination despite compliant HH gestures, including contamination from the glove box or through contact with the patient’s skin or handling of the tourniquet. The presence of microorganisms with a high ability to survive in the environment, such as *Acinetobacter*, *Enterococci* and *Bacillus cereus*, on the fingertips underscores the importance of maintaining a clean environment around the patient. We do not in any way advocate for the routine microbiological study of finger contamination among HCWs. However, we consider the findings from this specific study to be valuable for utilization in training sessions, as they effectively illustrate how HCWs’ fingers can become contaminated when HH practices are not optimal.

Using short videos depicting nurses failing to perform one or both of the required HH gestures during PVC placement procedures, along with the evaluation results of HH practices and the microbiological data obtained in the initial phase of the study, we have developed an educational tool specifically tailored to assist HCWs performing PVC insertions in comprehending the significance of adhering to HH and glove usage recommendations.

The participation of 48 hospitals in all four phases of the study provided with the opportunity to evaluate the impact of the training conducted with the developed tool on a representative sample of 280 HCWs. The training of these professionals with the educational tool led to a significant improvement in adherence to both HH practices and proper glove usage during PVC placement.

Our study has several limitations. Firstly, due to the demanding nature of the study, half of the centers did not continue their participation beyond the 1^st^ phase, which may have reduced the robustness of the impact study. However, the HCWs who were lost to follow-up and those whose practices could be studied before and after training, having been similar before training, we considered as a significant population of 280 HCWs. Secondly, the microbiological analysis was conducted using a single culture medium (Trypticase Soja sheep blood agar), which may have limited the growth of fastidious bacteria present on the hands of HCWs. However, since the objective of the study was to detect the pathogens tipically responsible for PVC-BSI (particularly *S. aureus* and *Enterobacteriales*), our results demonstrated that this objective was achieved. A third limitation was the two-month delay between the training and the second observation of PVC placement. The selection of a relatively brief time frame was deliberate, aimed at reducing the likelihood of losing subjects to follow-up and enabling the observation of the same HCWs both before and after training. This decision took into account the considerable turnover among HCWs in French hospitals. We investigated the reasons for the non-participation of some participants in the second (training) and third (practice observation after training) parts of the study. Several hypotheses can be formulated. The particular interest in microbiological sampling during the initial phase motivated a larger number of local teams. The imposition of a tight schedule for conducting the training may have discouraged or made it impossible for some teams to complete the training session. Lastly, conducting direct observations requires a significant commitment from the local team, and some teams were unable to allocate the necessary time for the second observation.

The national SPIADI team urged the local infection control teams to regularly conduct training sessions using our tool and to observe the trained HCWs to ensure the ongoing effectiveness of the training. A fourth limitation was the absence of observations for HCWs who did not undergo training, serving as controls.

## Conclusion

Our study demonstrated that training HCWs using our educational tool, which combines reminders of best practices and risk factors associated with PVC-related infections, engaging HCWs (presentation of practice evaluation), identifying professionals deviating from best practices (simulation videos), and objectively assessing fingertip contamination (microbiological study), significantly improved compliance with HH gestures and glove usage. We encourage infection control teams to utilize this tool to raise awareness among HCWs responsible for PVC placement about the risk of infection associated inadequate hand hygiene.

### Supplementary Information

**Additional file 1: Supplementary Figure 1.**

**Additional file 2: Supplementary Figure 2.**

## Data Availability

The datasets used and/or analysed during the current study are available from the corresponding author N. van der Mee-Marquet on reasonable request. The educational tool is available using this link https://www.spiadi.fr/tools?tab=1.
