# Supplementary material for: Impact of a training strategy on improving compliance of hand hygiene and gloving during the placement of a short peripheral venous catheter: the multicentre study CleanHand4
Source: BMC Med Educ. 2023 Oct 6;23:731. doi: 10.1186/s12909-023-04727-x (PMC10559517; doi:10.1186/s12909-023-04727-x)
Supplement: Supplementary file 1 — Additional file 1: Supplementary Figure 1. [file 12909_2023_4727_MOESM1_ESM.docx]

**Supplementary Figure 1.**

Standardized grid examining HH at the beginning of the procedure and immediately prior to PVC insertion.


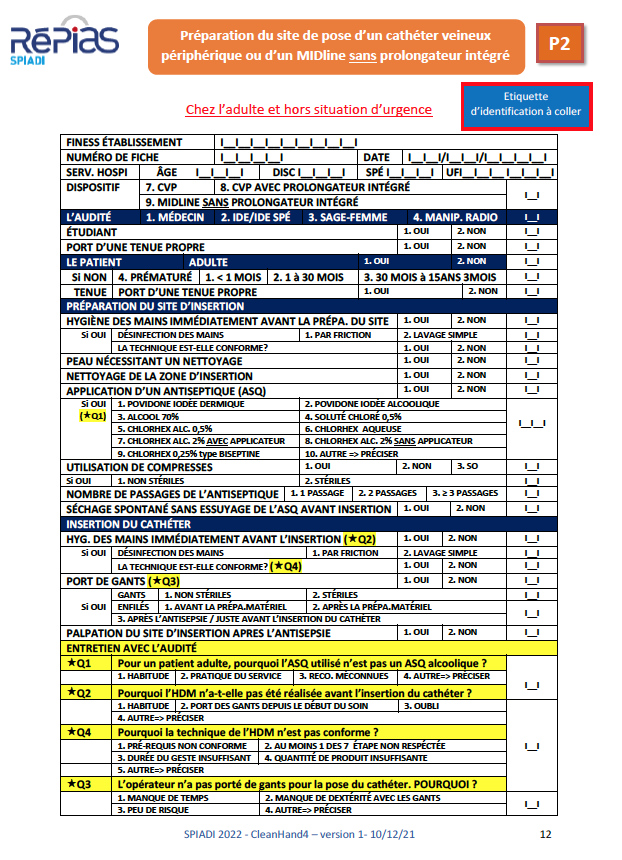


The Cleanhand4 study did not document the duration of care and any interruptions.
