# Supplementary material for: Impact of a training strategy on improving compliance of hand hygiene and gloving during the placement of a short peripheral venous catheter: the multicentre study CleanHand4
Source: BMC Med Educ. 2023 Oct 6;23:731. doi: 10.1186/s12909-023-04727-x (PMC10559517; doi:10.1186/s12909-023-04727-x)
Supplement: Supplementary file 2 — Additional file 2: Supplementary Figure 2. [file 12909_2023_4727_MOESM2_ESM.docx]

**Supplementary Figure 2.**

Regional distribution of the 91 participating centers.


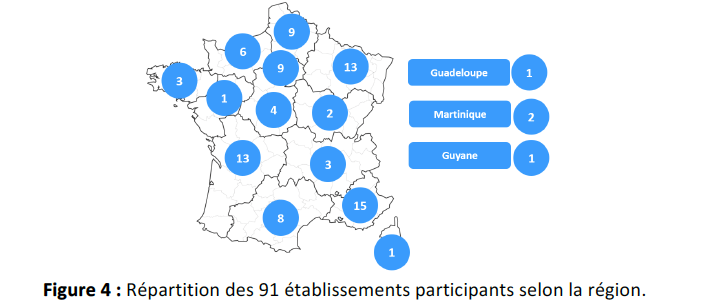


Currently, there are 2,695 healthcare facilities in France, which can be categorized as follows:

- 88 university hospitals
- 8 military hospitals
- 486 general hospitals (also known as secondary hospitals, equivalent to provincial hospitals, offering five to 10 clinical specialities such as haematology, oncology, nephrology, and intensive care)
- 567 short-stay clinics (healthcare facilities designed to provide medical evaluation, treatment, and care to patients with acute or minor medical conditions who do not require extended hospitalization)
- 18 oncology centers
- 118 dialysis centers
- 194 local hospitals (resembling primary hospitals, offering general medical services with few specialities, including internal medicine, obstetrics-gynaecology, paediatrics, general surgery, or general practice)
- 106 day centers
- 511 rehabilitation centers
- 104 psychiatric centers
- 495 long-stay hospitals.

Among the 2,695 facilities, 1,899 are part of the SPIADI network. These 1,899 hospitals have been invited to participate in the CleanHand4 study, regardless of their type (short-term or long-term stays). Information about the study is being disseminated through email and web conferences.
